# An unusual delusion of duplication in a patient affected by Dementia with Lewy bodies

**DOI:** 10.1186/s12883-017-0842-1

**Published:** 2017-04-19

**Authors:** Paolo Solla, Gioia Mura, Antonino Cannas, Gianluca Floris, Davide Fonti, Gianni Orofino, Mauro Giovanni Carta, Francesco Marrosu

**Affiliations:** 1 Department of Neurology, Movement Disorders Center, Institute of Neurology, University of Cagliari, SS 554 Bivio per Sestu, Monserrato, 09042 Cagliari Italy; 2 Department of Medical Sciences and Public Health, University of Cagliari, Cagliari, Italy; 3 Chair of Quality of Care and Applied Medical Technologies, Department of Public Health, Clinical and Molecular Medicine, University of Cagliari, Monserrato, Italy

**Keywords:** Dementia with Lewy bodies, Delusion of duplication, Misidentification syndromes, Capgras syndrome

## Abstract

**Background:**

Dementia with Lewy bodies (DLB) is the second most frequent diagnosis of progressive degenerative dementia in older people. Delusions are common features in DLB and, among them, Capgras syndrome represents the most frequent disturbance, characterized by the recurrent and transient belief that a familiar person, often a close family member or caregiver, has been replaced by an identical-looking imposter. However, other delusional conditions near to misidentification syndromes can occur in DLB patients and may represent a major psychiatric disorder, although rarely studied systematically.

**Case presentation:**

We reported on a female patient affected by DLB who presented with an unusual delusion of duplication. Referring to the female professional caregiver engaged by her relatives for her care, the patient constantly described the presence of two different female persons, with a disorder framed in the context of a delusion of duplication.

A brain 99Tc-hexamethylpropyleneamineoxime SPECT was performed showing moderate hypoperfusion in both occipital lobes, and associated with marked decreased perfusion in parieto-fronto-temporal lobes bilaterally.

**Conclusions:**

An occipital hypoperfusion was identified, although in association with a marked global decrease of perfusion in the remaining lobes. The role of posterior lobes is certainly important in all misidentification syndromes where a natural dissociation between recognition and identification is present. Moreover, the concomitant presence of severe attentional and executive deficits evocative for a frontal syndrome and the marked global decrease of perfusion in the remaining lobes at the SPECT scan also suggest a possible dysfunction in an abnormal connectivity between anterior and posterior areas.

**Electronic supplementary material:**

The online version of this article (doi:10.1186/s12883-017-0842-1) contains supplementary material, which is available to authorized users.

## Background

Dementia with Lewy bodies (DLB) is a progressive degenerative dementia, with core features characterized by fluctuating cognitive symptoms with pronounced variations in attention and alertness, recurrent visual hallucinations and associated features of parkinsonism [1], which is recognized as the second most common form of severe cognitive impairment in older people [2].

Delusions are common features in DLB, and patients affected by this condition are firmly hold on their false beliefs also in the presence of strong contradictory evidence. Among delusional disturbances in DLB, Capgras syndrome represents the most frequent disorder affecting approximately 17% of DLB patients and is characterized by the recurrent and transient belief that a familiar person, often a close family member or caregiver, has been replaced by an identical-looking imposter [3]. Capgras syndrome belongs to delusional misidentification syndromes (see Additional file 1: Table S1), among which two major groups were proposed, based on content-specific misidentifications [4, 5]: the Capgras type (also including the Fregoli delusion, a delusional belief that one or more familiar persons, usually persecutors following the patient, are masquerading as several other people) and the Clonal Pluralization type. In the last, patients firmly belief that multiple exact copies of places, objects, self or others exist, such as in the misidentification of reflection, in the reduplicative paramnesia, and in the clonal pluralization of the self [6–8]. However, other delusional conditions near to misidentification syndromes can occur in several patients with DLB and may represent a major psychiatric disorders, although rarely studied systematically. Here we reported on a female patient affected by DLB who presented with an unusual delusion of duplication.

## Case presentation

A 77-year-old woman had a history of DLB since 2013 when she was 75. She has a primary school education and was a retired saleswoman. Previous clinical history was unremarkable, she did not assume any medication, and there was no family history of psychiatric or movement disorders. Her initial motor symptoms, started approximately six months before the first examination, were bilateral bradykinesia, followed by rigidity and mild postural instability with associated marked hypomimia and mild hypophonia. Her relatives signaled also a history of likely rapid eye movement sleep behavior (RBD) disorder. Mood and affect were normal, with the exception of mild symptoms of apathy described mainly as a lack of motivation. She was initially treated with levodopa/carbidopa 400/100 mg daily with good initial improvement of parkinsonian symptoms. In agreement with the patient, no medications were introduced to treat RBD symptoms, which were considered not significant by the same patient.

However, three months later, she noticed a moderate increase of her motor symptoms and selegiline 10 mg daily was added. After another month, we were contacted by her relatives because she has started to present a significant cognitive impairment with fluctuating episodes of confusion and visual hallucinations, and, although less marked, with delusional jealousy and persecutory ideas, sometimes with behavioral problems (aggressiveness). Brain MRI was normal and without significant atrophy. Her dementia work-up did not reveal any reversible causes, while results of laboratory tests were normal. Although clinical feature were not suggestive for prion disease or epilepsy, an EEG was done and was normal.

**Fig. 1:**
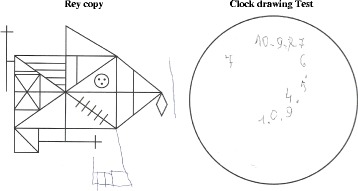
Rey Copy and the Clock drawing Test showing the severe visuospatial dysfunction

**Fig. 2:**
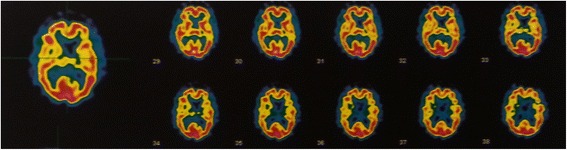
Brain ^99^mTc-hexamethylpropyleneamineoxime single-photon emission computed tomography (SPECT) imaging shows moderate hypoperfusion in both occipital lobes, and associated with marked decreased perfusion in parieto-fronto-temporal lobes bilaterally. SPECT images were generated in the transaxial plane, with direction of the slices from feet to head

A neuropsychological evaluation was done (Table 1) which showed a moderate-severe cognitive impairment mainly characterized by severe attention and executive deficits and frontal syndrome, with associated severe deficit of visual-spatial functions. Rey Copy and the Clock drawing tests are reported in Fig. 1. Moderate/severe impairment in long-term verbal and visual-spatial memory recall was registered with deficit of fluency for semantic categories (mainly due to attentional fluctuations). A brain 99Tc-hexamethylpropyleneamineoxime single-photon emission computed tomography (SPECT) was performed showing moderate hypoperfusion in both occipital lobes, and associated with marked decreased perfusion in parieto-fronto-temporal lobes bilaterally (Fig. 2). At this time, she was diagnosed as DLB.

**Table 1:** Neuropsychological findings of the subject at evaluation

Test	Raw score	Adjusted score and/or (Cut-off)	Equivalent score and/or classification
MMSE	18/30	17.7 (>24)	Impaired
ADL	3/6		Impaired
IADL	3/6		Impaired
Frontal assessment battery	10/18	11.5 (>13.4)	0
Stroop Test			
Time Interference	93.5	80.25 (<36.91)	0
Error interference	15	13 (<4.23)	0
Attentional matrices	25	30.75 (>31)	0
Trail making test	NP		Impaired
Digit span forward	4	4,65 (>4.26)	2 - Normal
Digit span backward	3	3.77 (>2.66)	2- Normal
Rey Auditory Verbal Learning Test			
Learning	27/75	37 (>28.53)	3 - Normal
Recall	0/15		0
Recognition (False recognition)	15/15 (6)		Impaired
Test of Corsi			
Direct	2	2.68 (>3.46)	0 - Impaired
Forward	3	3.42 (>3.08)	2 - Normal
Phonological verbal fluency	31	40 (>16)	4 - Normal
Semantic verbal fluency	10	20 (>25)	0 - Impaired
Modified Card Sorting Test (MCST)	NP		Impaired
Rey figure (Copy)	0	(>23.74)	0 - Impaired
Immediate recall	1	8.6 (>6.44)	1 -At lower limit
Delay recall	0	(>6.33)	0 - Impaired-
Simple Figure Copy	3	4.3 (>7.18)	0 - Impaired
Clock Test	0	>3	Impaired

*Legend*. Raw score, score test; Adjusted score: obtained by subtracting or adding the contribution of patient’s age and education; Equivalent score: adjusted scores converted to a five-point interval scale, ranging from 0 to 4 equivalent scores. Score 0 was equal or lower than the outer tolerance limit (5%), NP, not performable

Thus, delusional jealousy and persecutory ideas were treated with the simultaneous suspension of selegiline and the introduction of quetiapine 12,5 mg/daily. In the following two weeks, both disappearance of these delusions and the normalization of behavioral problems were observed, although a moderate impairment of bradkynesia and rigidity was noted.

However, after four months, her family members reported the appearance of an unusual delusion, characterized by the fact that the patient, referring to the female professional caregiver engaged by her relatives for her care, constantly described the presence of two different female persons, sometimes with different physical characteristics, sometimes similar, with a disorder framed in the context of a delusion of duplication. It was reported, in fact, that the patient always talked about the "two ladies", despite the complaints of family members who indicated the obvious presence of a single person, without substantial criticism of the delusional disorder by the patient. Also in the absence of the professional caregiver, the aberrant conviction of the existence of two different ladies was constantly present, while the caregiver was never recognized as a unique person by the patient. In this respect, during the conversation the patient said: “Today I have arrived to the hospital with my son, without the two ladies who usually take care of me.”, “They are nice people, even if they do not speak much. I do not remember their names, but both speak with a foreign accent”. When we asked if they were both with blond hair, her response was: “I do not remember exactly the color of their hair, but it seems to be of a different color”.

Thus, levodopa was decreased to 200 mg/daily, with no disappearance of the delusion of duplication, but with a clear worsening of parkinsonism. At a follow-up visit, two months later, with the same professional caregiver present during the interview, the patient was referring to her as if it actually were two people. The delusional disorder of duplication was not present in relation to other people or other patient’s relatives. No visual or auditory hallucinations were referred.

## Discussion

Here, we have described a particular delusion of duplication quite different both to the classical Capgras syndrome described in DLB and to the typical clonal pluralization syndromes. In fact, the delusion was not characterized by the patients’s belief that her professional caregiver was been replaced by an identical imposter, but, on the contrary, by the duplication of the same person. Furthermore, in Capgras syndrome, the imposter commonly has features that are very similar to those of the original person, although subtle physical differences are used to differentiate the original person from the imposter. In our case, according the description made by the patient herself, the core of the delusional idea was not characterized by the perception of an imposter, but was simply described as a phenomenon of reduplication. This delusion was not simply an unusual psychotic symptom driven uniquely by medication, because an important reduction of levodopa/carbidopa dosage did not change its clinical presentation, while a total suspension of dopaminergic treatment was impossible for the worsening of parkinsonian symptoms.

Another striking difference with Capgras syndrome is represented by the person involved in the appearance of this delusion of duplication. In fact, while in Capgras syndrome, patients report that one or more well-known persons (usually family members) have been replaced by substitutes [4], it is interesting to note that this delusion of duplication was referred only to the professional caregiver and not to family members. In this context, it should be considered as the caregiver is at the same time both the nearest and the more extraneous person. On the other hand, the clonal pluralization syndromes are characterized by the false belief of the existence of an identical copy (clone) of the patient’s self or of other individuals; in our case, the patient believed that there was another caregiver in addition to that real, but she did not refer to her as an exact copy, rather than as she was a different individual. This particular form of reduplicative delusion could explain why the patient did not show persecutory ideas neither toward the real caregiver nor the “other” caregiver: simply, she believed to have two caregivers.

This phenomenon of reduplication appears very interesting because its similarity to a type of hallucinatory disorder raises the question on its clear operating framework between these psychiatric conditions. However, the previous presence of other delusional disturbances and the fact that this belief, with a duration longer than two months, was fixed and not amenable to change in light of conflicting evidence, in addition to the absence of any other hallucinatory problem, depose with greater evidence to a classification within the spectrum of delusions, according to the DSM 5 [9]. In any case, it should be remembered as a previous study found a strong relationship between Capgras syndrome and visual hallucinations [3].

Another issue concerns the possible neurobiological mechanisms that may contribute to the appearance of this delusion of duplication. In fact, several studies have addressed their attention to Capgras syndrome, but other misidentification syndromes have been scarcely studied. With regard to Capgras syndrome, proposed alterations include the presence of impairment in facial processing [10, 11], dysfunction of working memory [12], altered connectivity among associative areas and limbic/paralimbic structures [13], bilateral dysfunction of fronto-temporal connectivity [14, 15], and right hemispheric hypo function [16, 17].

In our patient with this peculiar delusion of duplication, an occipital hypoperfusion was identified, although in association with a marked global decrease of perfusion in the remaining lobes. In this context, it should be kept in mind as a deficiency in occipital hypoperfusion is more frequently seen in DLB with respect to other dementia such as Alzheimer disease [18]. The role of posterior lobes is certainly important in all misidentification syndromes where a natural dissociation between recognition and identification is present and is confirmed by the findings of severe deficit of visual-spatial functions observed at the neuropsychological evaluation and typical of a posterior cerebral areas dysfunction. However, the concomitant presence of severe attentional and executive deficits evocative for a frontal syndrome and the marked global decrease of perfusion in the remaining lobes at the SPECT scan also suggest a possible dysfunction in an abnormal connectivity between anterior and posterior areas.

Moreover, the presence of a significant impairment in visual-spatial functions and in visual-spatial memory recall is in agreement with previous neuropsychological findings on delusional misidentifications reporting a low efficiency in the complex visuospatial organization tasks and in non-verbal memory [19]. In this context, there is a clear evidence for impaired visuoperceptual functions in the appearance of misidentificative psychotic symptoms in patients affected by Alzheimer's disease [20].

## Conclusions

In conclusion, we have described an unusual delusion of duplication in a patient affected by LBD. Neural correlates of this peculiar delusion of duplication in LBD still remain largely unclear, and future researches are required to better identify possible neurobiological mechanisms that may contribute to the appearance of this peculiar type of delusion.

## Additional file

Additional file 1: Table delusions of misidentification. Table summarizing the most common delusions of misidentification and related delusions. (DOCX 483 kb)

## Abbreviations

DLB: Dementia with Lewy bodies
MRI: Magnetic resonance imaging
RBD: Rapid eye movement sleep behavior
SPECT: Single-photon emission computed tomography

## References

[1] McKeith IG, Dickson DW, Lowe J, Emre M, O'Brien JT, Feldman H (2005). Diagnosis and management of dementia with Lewy bodies: third report of the DLB Consortium. Neurology.

[2] Vann Jones SA, O'Brien JT (2014). The prevalence and incidence of dementia with Lewy bodies: a systematic review of population and clinical studies. Psychol Med.

[3] Thaipisuttikul P, Lobach I, Zweig Y, Gurnani A, Galvin JE (2013). Capgras syndrome in Dementia with Lewy Bodies. Int Psychogeriatr.

[4] Murai T, Toichi M, Yamagishi H, Sengok A (1998). What is meant by ‘misidentification’ in delusional misidentification syndromes. Psychopathology.

[5] Salvatore P, Bhuvaneswar C, Tohen M, Khalsa HM, Maggini C, Baldessarini RJ (2014). Capgras' syndrome in first-episode psychotic disorders. Psychopathology.

[6] Politis M, Loane C (2012). Reduplicative paramnesia: a review. Psychopathology.

[7] Voros V, Tényi T, Simon M, Trixler M (2003). “Clonal pluralization of the self”: a new form of delusional misidentification syndrome. Psychopathology.

[8] Ranjan S, Chandra PS, Gupta AK, Prabhu S (2007). Clonal pluralization of self, relatives and others. Psychopathology.

[9] The Diagnostic and Statistical Manual of Mental Disorders (5th ed.; DSM–5; American Psychiatric Association, 2013)

[10] Walther S, Federspiel A, Horn H, Wirth M, Bianchi P, Strik W, Muller TJ (2010). Performance during face processing differentiates schizophrenia patients with delusional misidentifications. Psychopathology.

[11] Phillips ML, David AS (1995). Facial processing in schizophrenia and delusional misidentification: cognitive neuropsychiatric approaches. Schizophr Res.

[12] Papageorgiou C, Lykouras L, Ventouras E, Uzunoglu N, Christodoulou GN (2002). Abnormal P300 in a case of delusional misidentification with coinciding Capgras and Fregoli symptoms. Prog Neuropsychopharmacol Biol Psychiatry.

[13] Weinstein EA (1994). Classification of delusional misidentification syndromes. Psychopathology.

[14] Joseph AB, O'Leary DH, Wheeler HG (1990). Bilateral atrophy of the frontal and temporal lobes in schizophrenic patients with Capgras syndrome: a case-control study using computed tomography. J Clin Psychiatry.

[15] Feinberg TE, Roane DM (2005). Delusional misidentification. Psychiatr Clin North Am.

[16] Cutting J (1991). Delusional misidentification and the role of the right hemisphere in the appreciation of identity. Br J Psychiatry Suppl.

[17] Weinstein EA, Burnham DL (1991). Reduplication and the syndrome of Capgras. Psychiatry.

[18] Lobotesis K, Fenwick JD, Phipps A, Ryman A, Swann A, Ballard C, McKeith IG, O'Brien JT (2001). Occipital hypoperfusion on SPECT in dementia with Lewy bodies but not AD. Neurology.

[19] Paillère-Martinot ML, Dao-Castellana MH, Masure MC, Pillon B, Martinot JL (1994). Delusional misidentification: a clinical, neuropsychological and brain imaging case study. Psychopathology.

[20] Reeves SJ, Clark-Papasavas C, Gould RL, Fytche D, Howard RJ (2015). Cognitive phenotype of psychotic symptoms in Alzheimer's disease: evidence for impaired visuoperceptual function in the misidentification subtype. Int J Geriatr Psychiatry.

