# Supplementary material for: An unusual delusion of duplication in a patient affected by Dementia with Lewy bodies
Source: BMC Neurol. 2017 Apr 19;17:78. doi: 10.1186/s12883-017-0842-1 (PMC5395768; doi:10.1186/s12883-017-0842-1)
Supplement: Additional file 1: — Table delusions of misidentification. Table summarizing the most common delusions of misidentification and related delusions. (DOCX 483 kb) [file 12883_2017_842_MOESM1_ESM.docx]

**Table summarizing the most common delusions of misidentification and related delusions.**

| Name | Definition |
| --- | --- |
| Capgras Syndrome | Recurrent and transient belief that a familiar person, often a close family member or caregiver, has been replaced by an identical-looking imposter. |
| Fregoli Syndrome | A delusional belief that one or more familiar persons, usually persecutors following the patient, are masquerading as several other people. |
| **Clonal Pluralization type Syndromes** | Belief that multiple exact copies of places, objects, self or others exist, such as in reduplicative paramnesia, and in the clonal pluralization of the self. |
| Syndrome of subjective doubles | Belief that familiar or unfamiliar people are physically and mentally transformed into the patient. |
| Mirrored self- misidentification | Belief that one’s reflection in a mirror is another person. |
| Cotard Syndrome | A nihilistic delusional belief ranging from the fixed and unshakable delusion that one is dead, does not exist or has lost internal organs, body parts, blood, or one’s soul. |
